# Exercise-Induced Bronchospasm and Atopy in Ghana: Two Surveys Ten Years Apart

**DOI:** 10.1371/journal.pmed.0040070

**Published:** 2007-02-27

**Authors:** Emmanuel O. D Addo-Yobo, Ashley Woodcock, Adorkor Allotey, Benjamin Baffoe-Bonnie, David Strachan, Adnan Custovic

**Affiliations:** 1 Department of Child Health, Komfo Anokye Teaching Hospital, Kumasi, Ghana; 2 North West Lung Centre, Wythenshawe Hospital, University of Manchester, Manchester, United Kingdom; 3 Department of Public Health Sciences, St George's Hospital Medical School, London, United Kingdom; National Heart and Lung Institute, United Kingdom

## Abstract

**Background:**

Asthma and allergic diseases have increased in the developed countries. It is important to determine whether the same trends are occurring in the developing countries in Africa. We aimed to determine the time trend in the prevalence of exercise-induced bronchospasm (EIB) and atopic sensitisation over a ten-year period in Ghanaian schoolchildren.

**Methods and Findings:**

Two surveys conducted using the same methodology ten years apart (1993 and 2003) among schoolchildren aged 9–16 years attending urban rich (UR), urban poor (UP), and rural (R) schools. Exercise provocation consisted of free running for six minutes. Children were skin tested to mite, cat, and dog allergen. 1,095 children were exercised in 1993 and 1,848 in 2003; 916 were skin tested in 1993 and 1,861 in 2003. The prevalence of EIB increased from 3.1% (95% CI 2.2%–4.3%) to 5.2% (4.3%–6.3%); absolute percentage increase 2.1% (95% CI 0.6%–3.5%, *p* < 0.01); among UR, UP, and R children EIB had approximately doubled from 4.2%, 1.4%, and 2.2% to 8.3%, 3.0% and 3.9% respectively. The prevalence of sensitisation had also doubled from 10.6%, 4.7%, and 4.4% to 20.2%, 10.3%, and 9.9% (UR, UP, and R respectively). Mite sensitisation remained unchanged (5.6% versus 6.4%), but sensitisation to cat and dog increased considerably from 0.7% and 0.3% to 4.6% and 3.1%, respectively. In the multiple logistic regression analysis, sensitisation (odds ratio [OR] 1.77, 95% CI 1.12–2.81), age (OR 0.88, 95% CI 0.79–0.98), school (the risk being was significantly lower in UP and R schools: OR 0.40, 95% CI 0.23–0.68 and OR 0.54, 95% CI 0.34–0.86, respectively) and year of the study (OR 1.73, 95% CI 1.13–2.66) remained significant and independent associates of EIB.

**Conclusions:**

The prevalence of both EIB and sensitisation has approximately doubled over the ten-year period amongst 9- to 16-year-old Ghanaian children irrespective of location, with both EIB and atopy being more common among the UR than the UP and R children.

## Abstract 2

The prevalence of both exercise-induced bronchospasm and sensitisation has approximately doubled over the ten-year period amongst 9- to 16-year-old Ghanaian children

## Editors' Summary

**Background.:**

The proportion of children with asthma is thought to be increasing worldwide, and particularly among children that live in more developed countries. However, it is not clear why this is, since many different aspects of lifestyle and the environment have been linked with the onset of asthma. In Africa, asthma has typically been thought of as being very uncommon, and indeed in many African dialects there is no word for asthma or the symptoms, such as wheezing, that asthmatic children experience. However, some research studies have suggested that asthma might be becoming more common in Africa and that this could be linked to ongoing economic and social changes.

**Why Was This Study Done?:**

The researchers here wanted to understand whether the trend for childhood asthma to be on the increase worldwide was also the case in Africa. Economic growth is bringing about rapid changes in lifestyle in many developing countries, and at the same time the burden of disease is changing. In order to make sure that health systems are appropriately resourced, it's important to anticipate future changes in the burden of different diseases.

**What Did the Researchers Do and Find?:**

This study was based on a comparison between two surveys, carried out ten years apart, of children attending three schools in Ghana's second largest city, Kumasi. The surveys were done in 1993 and 2003, and the schools surveyed were a rich city school, a poor city school, and a school in the nearby countryside. The same methods were used in the two different surveys. Importantly, the researchers used an exercise test as an indicator for asthma, because language differences meant they could not find out whether children were indeed asthmatic. In the exercise test, the schoolchildren ran outdoors for six minutes, and the researchers measured how fast the children could breathe out before and after exercise (their “peak flow”). Children whose drop in peak flow was more than 12.5% were classified as having exercise-induced bronchospasm, which is thought to predict asthma. The children were also tested for their response to extracts that commonly cause allergic reactions, such as from dust mites and cat and dog hair. 1,095 children were studied in 1993 and 1,848 in 2003, paralleling the growth of the city, which also meant that by 2003 the rural school had become incorporated into the city. Over this period of time, the proportion of children with exercise-induced bronchospasm increased in all three schools; overall this proportion went up from 3.1% to 5.2%. Children from the rich city school were most likely to have exercise-induced bronchospasm at either survey date. However, children from the poor city school experienced the biggest change over the time period studied, with more than double the proportion of children having exercise-induced bronchospasm in 2003 as compared to 1993. The researchers also saw similar trends in children who had allergic reactions to common substances.

**What Do These Findings Mean?:**

The researchers observed substantial increases in the rate of exercise-induced bronchospasm, and allergic reactions, between the two survey dates. This finding suggests that asthma is likely to have become much more common in that time. However, exercise-induced bronchospasm is not an exact indicator of asthma so it is not possible to be certain about this. These changes are likely to be linked with the adoption of westernized lifestyles, but which precise factors are responsible for the increase is not clear. Factors linked to the development of asthma include a lower rate of childhood infections, a lower rate of breast-feeding, environmental pollution, and many others. Links between the increase in exercise-induced bronchospasm and any of these factors were not examined in this study. However, these results suggest that if the findings here are common to other African cities as well, a greater proportion of African health budgets will need to be devoted to asthma care in the future.

**Additional Information.:**

Please access these Web sites via the online version of this summary at http://dx.doi.org/10.1371/journal.pmed.0040070.

- Wikipedia has an entry on asthma (Wikipedia is an internet encyclopedia anyone can edit)
- The World Health Organization's Ghana minisite has information on this country
- Patient information from NHS Direct on asthma
- An accompanying *PLoS Medicine* Essay by Matthias Wjst and Daniel Boakye discusses research on asthma in Africa

## Introduction

Early reports from developing countries in Africa over three decades ago suggested that childhood asthma was uncommon and not associated with atopic sensitisation [1–6]. However, due to cultural and linguistic differences in this region it is often difficult to adopt a universally accepted asthma definition and accurately compare the findings between different areas. This problem is emphasised by the fact that there is no word for wheeze or asthma in most of the local dialects. To circumvent this, exercise-induced bronchospasm (EIB) may be used as an objective indicator of asthma [7], although its value may be limited by environmental factors (e.g., temperature, humidity, air pollution) [8].

Using EIB as a marker of asthma, several recent cross-sectional reports suggested that asthma appears common among African children [9–13]. These studies also reported relatively high prevalence of atopic sensitisation (previously uncommon in these countries), and an increasing association between asthma and sensitisation [9–12]. Furthermore, a higher prevalence of asthma and atopy was observed among urban than rural children, and in the more affluent communities [9–12,14]. These findings indirectly suggest that asthma prevalence may be increasing in Africa. As African countries experience economic growth, social conditions and lifestyles are likely to change, transforming the pattern of diseases [15]. It is therefore important to determine whether the suggested increase in asthma prevalence in Africa is real, as the cost of treatment could overwhelm health budgets.

In 1993, we conducted a study among Ghanaian schoolchildren to determine the prevalence of EIB and atopic sensitisation [11]. In the current study, we aimed to determine the change in the prevalence of EIB and atopy over the subsequent ten-year period.

## Methods

### Setting

We conducted two surveys in the same setting using exactly the same methodology ten years apart (1993 [11] and 2003) in Kumasi, Ghana, among children aged 9–16 years during the dry season (February–March). The study was approved by the Ghana Education Service and the management of the University of Science and Technology Primary and Junior Secondary Schools, Kumasi, and parental consent was obtained through the heads of the schools.

### Participants

In the 1993 surveys, we categorised schools in Kumasi into urban poor (UP) and urban rich (UR) according to the fees charged [11]. One school from each category was selected based on proximity (Kotei Primary and Junior Secondary schools from the UP category, University of Science and Technology Primary and Junior Secondary schools from the UR category), and a third school from a nearby rural area was selected on the basis of size and accessibility (Ohwim Primary and Junior Secondary schools). The UR and UP schools were situated two kilometres apart and the rural (R) school was situated 20 kilometres outside Kumasi. We used the same schools in 2003. To ensure consistency, all tests were performed by the same investigators in both surveys.

### Procedures

#### Skin testing.

Children were skin-prick tested to the common inhalant allergens (*Dermatophagoides pteronyssinus, D. farinae,* cat, and dog, with positive and negative controls; Allergopharma, http://www.allergopharma.de). Sensitisation was defined as wheal at least 3 mm greater than the negative control. In order to ensure that the potency of the diagnostic solutions in 2003 was comparable to that in 1993, major allergen content of the skin testing extracts was determined using two-site monoclonal antibody-based ELISAs (Der p 1 for *D. pteronyssinus,* Der f 1 for *D. farinae,* Fel d 1 for cat, and Can f 1 for dog); the results confirmed batch-to-batch consistency.

#### Exercise test.

In 1993 we investigated 200 healthy participants and defined a normal response to exercise challenge as a 12.5% or less postexercise drop in peak expiratory flow rate (PEFR) [11]; this value was used as a cutoff for positive exercise test.

After their height and weight were measured, each child was assigned his/her own peak-flow meter and its use was demonstrated to the whole group. The children practised until good technique was achieved (three readings less than 5% apart). At the beginning of the test PEFR was recorded (the best of three consecutive blows) and compared with age/height-predicted data. An exercise challenge was not performed if PEFR was below 60% of predicted.

The exercise challenge consisted of free running outdoors for 6 min, aiming at a heart rate of more than 170 beats/min or 85% maximum for age (whichever was greater). The PEFR manoeuvre was repeated 5 min and 8 min after exercise, and the lower PEFR was recorded.

Tests were carried out between 0800 and 1200 hours. Relative humidity was recorded as the mean between those recorded at 0600 and 1200 hours, and temperature as the daily mean.

### Statistical Analysis

Statistical analysis was carried out using SPSS 11.0 (http://www.spss.com) and confidence interval analysis programmes. Wilson's method was used to calculate means and 95% confidence intervals (CIs) for prevalences and for their differences between years [16]. We used χ^2^ test to determine the significance of differences in categorical variables, and unpaired t-tests for continuous variables. We calculated CIs for estimates of relative risks (RRs) for the outcomes in 2003 compared to 1993 [16]. The relationship between EIB and risk factors including age, gender, body mass index (BMI), school, sensitisation, and year of the study was analyzed using multiple logistic regression. Odds ratios (ORs) were estimated using the regression models, and 95% CIs were generated according to Wald, using a *p*-value of 0.05 as significant.

## Results

All children aged 9–16 y attending the schools participated in the study. A total of 1,095 children (557 [50.9%] male) were exercised in 1993 (599 UR, 220 UP, and 276 R) and 1,848 (935 [50.6%] male) in 2003 (651 UR, 559 UP, and 638 R); 916 parents and children consented to skin testing in 1993 and 1,861 in 2003. In 1993, one child in the rural school was not allowed to run because of severe sickle cell anaemia. No child was excluded because of low baseline PEFR. In 2003, a total of 16 children (4 UR, 5 UP, 7 R) were excluded from exercise test response analysis because they failed to complete the 6-min free running exercise. None of them had an abnormal baseline PEFR.

There was little difference in the environmental factors between the two study periods (1993 and 2003, temperatures 28 °C and 29 °C and relative humidities 60% and 71%, respectively).

Demographic characteristics of the study populations are presented in Table 1. Participants in 2003 were slightly older than those in 1993 (average about 6 mo). In 1993, children from the UP and R schools had similar weights and heights, whereas children from the UR school were significantly heavier and taller. In 2003 weights and heights of children from the three schools differed significantly, with UR children being heaviest and tallest and R children lightest and shortest (Table 1). In 2003, children in all three schools were significantly heavier and taller compared to 1993.

**Table 1:**
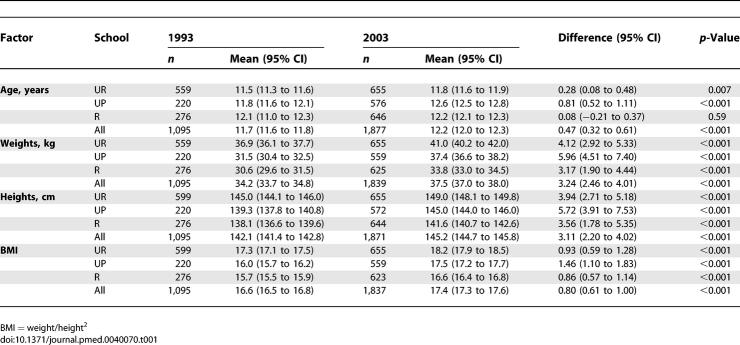
Demographic Characteristics of the Study Populations in 1993 and 2003

### EIB and Atopic Sensitisation, 1993–2003

The prevalence of EIB, atopic sensitisation, and sensitisation to individual allergens in 1993 and 2003 is presented in Table 2. Overall, prevalence of EIB had increased from 3.1% (95% CI 2.2%–4.3%) in 1993 to 5.2% (95% CI 4.3%–6.3%) in 2003 (absolute percentage increase 2.1%, 95% CI 0.6%–3.5%, *p* = 0.009). The prevalence of EIB had approximately doubled from 4.2%, 1.4%, and 2.2% in 1993 to 8.3%, 3.0%, and 3.9% in 2003 (UR, UP, and R schools respectively; Table 2). Although the pattern was similar in all three schools, this change was statistically significant only in the UR school and the whole sample (Table 2). As in the 1993, EIB in 2003 was substantially more prevalent among the UR compared to UP or R children (*p* < 0.001).

**Table 2:**
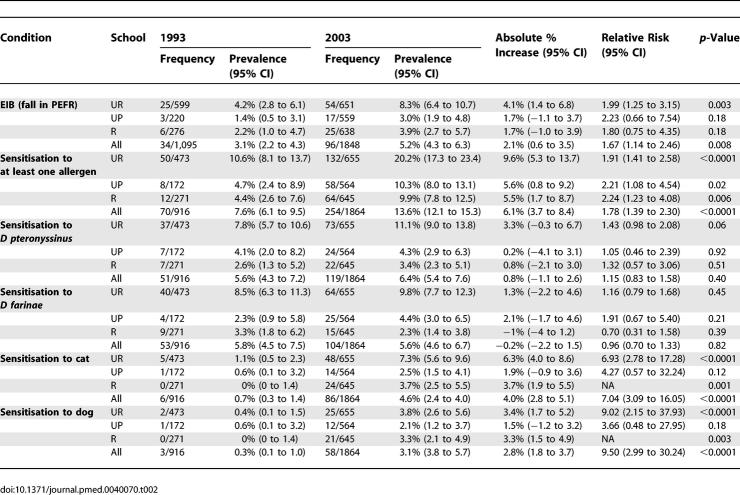
Changes in Prevalence of Exercise-Induced (Bronchospasm) Fall in PEF and Sensitisation in Ghanaian Schoolchildren Aged 9–16 Years, 1993 to 2003

The prevalence of sensitisation had approximately doubled from 10.6%, 4.7%, and 4.4% in 1993 to 20.2%, 10.3%, and 9.9% in 2003 (UR, UP, and R respectively, Table 2). While the highest prevalence was observed in the UR children, the largest changes were observed in the R and UP schools. In both surveys the prevalence of sensitisation was similar in UP and R schools, and significantly lower than in the UR school.

Sensitisation to dust mites *(D. farinae, D. pteronyssinus)* remained common, but unchanged over the 10-y period (5.8% and 5.6% in 1993, 5.6% and 6.4% in 2003). However, sensitisation to cat and dog, which was very rare in 1993 (0.7% and 0.3%, respectively) increased considerably, to 4.6% and 3.1% in 2003.

### Relationship between Atopic Sensitisation and EIB

The proportion of EIB-positive children who were atopic had increased from 13.3% in 1993 to 24% in 2003. In 1993 there was no significant association between sensitisation and EIB (OR 1.9, 95% CI 0.65–5.64, *p* = 0.24). In 2003 sensitisation significantly increased the risk of EIB (OR 2.10, 95% CI 1.29–3.42, *p* = 0.003).

In the multiple logistic regression analysis including age, gender, BMI, school, sensitisation, and year of the study, the following variables remained significant and independent associates of EIB: atopic sensitisation (OR 1.77, 95% CI 1.12–2.81, *p* = 0.015), age (OR 0.88, 95% CI 0.79–0.98, *p* = 0.02), school (using UR school as a reference, the risk of EIB was significantly lower in UP and R schools [OR 0.40, 95% CI 0.23–0.68, *p* = 0.001; OR 0.54, 95% CI 0.34–0.86, *p* = 0.009, respectively]), and year of the study (OR 1.73, 95% CI 1.13–2.66, *p* = 0.01).

## Discussion

### Principal Findings

To our knowledge this is the first study in Africa to compare objective measures of atopic sensitisation and exercise-induced airway reactivity suggestive of asthma over a ten-year period. The prevalence of both EIB and sensitisation has increased substantially amongst Ghanaian schoolchildren. Sensitisation to pet allergens, which was uncommon in 1993, is becoming considerably more frequent. This observation was made in association with contemporaneous improvements in nutritional status, with an increase in weight, height, and BMI. The biggest changes in EIB prevalence were observed in the UP school, whereas the biggest change in atopy was observed in the UP and R schools. However, EIB and atopy were still more common among UR than among UP and R children.

### Strengths and Weaknesses of the Study

Both surveys were conducted at the same time of year using identical methods, allowing objective comparisons. It is worth emphasising that, in marked contrast to 1993, rapid growth in the city population (from 700,000 to 1.3 million) made the “rural” setting in 2003 almost indistinguishable from the “urban” in terms of infrastructure and lifestyle. Ghana has a high national population growth rate of about 2% per annum, as a result of high fertility and declining infant mortality rates (which improved from 69/1,000 live births in 1993 to 55/1,000 live births in 2005, probably due to improved access to health care). This growth rate, together with rural-to-urban migration in search of better jobs, may explain the rapid population growth in the city. Consequently, enrolment in the UP and R schools increased, which was reflected in a considerably larger sample size and the fact that in the 2003 survey children were slightly older. However, the increase in the number of children recruited in 2003 compared to 1993 is unlikely to have affected the main outcomes.

Environmental pollution increased as a consequence of increased economic activity. While this factor may have affected exercise test responses, the extent to which it did is difficult to estimate, although it is likely to be minimal.

It should be emphasised that, although EIB is suggestive of asthma, these conditions are not equivalent, and asthma can be present in the absence of positive exercise challenge test, and vice versa. Airway hyper-responsiveness has been extensively assessed as an objective marker of asthma, since it is an important feature of the disease and is relatively easy to measure. Exercise is one of the most common triggers of brief asthma symptoms, and seems to be a specific trigger for patients with asthma, since it rarely causes bronchoconstriction in normal individuals or those with other respiratory conditions. The mechanisms by which exercise leads to airflow limitation are not completely understood, but there are two proposed theories (the hyperosmolarity theory and the airway rewarming theory, which refer, respectively, to changes in the osmolarity of the fluid lining of the lung and to the cooling and rewarming of the airway mucosa) [17,18].

### Meaning of the Study

In contrast to studies in economically developed countries that have suggested that asthma and atopy epidemics have reached a peak or may have declined in the last decade [19], our study suggests that the increase in prevalence in Africa is continuing. Policy-makers should take this into account, as the high cost of asthma treatment may significantly affect health budgets of developing countries in coming decades.

Factors associated with the transition from rural to urban lifestyles could be responsible for the observed increases in EIB and atopy in this community. Notable differences between the traditional and “westernised” lifestyles in Ghana include diet (fresh versus canned and processed food with higher salt and fat content) and environment (outdoor living with exercise versus sedentary indoor living). Although affluent urban Ghanaian children are more prone to obesity than rural children, the increased prevalence of EIB cannot be explained by obesity [20,21]. In our study, very few of the children would be classified as obese. Children in 2003 survey were slightly older, but the increase in size could not be fully explained by the six-month age difference between two surveys. A significant increase in weight, height, and BMI was observed amongst rural children, and there was no difference in age in this group.

Improved living standard, diet, and nutritional status may result in fewer infections, which could have contributed to the observed differences. Urban children tend to have fewer infections of narrower scope than do rural children in Ghana. However, malaria is common in both urban and rural areas, and our earlier studies showed that helminthic infection are uncommon in this area due to habitual use of anthelmintic medication [22].

The rate of increase in sensitisation to cat and dog in this study population is remarkable. It is worth emphasising that we have checked the potency of the allergenic extracts used for skin testing in 1993 and 2003 by measuring the major allergen content, and have found them to be comparable. In a recent study of risk factors for asthma in Ghanaian children, we found that despite a high rate of pet ownership (comparable to the UK), pet allergen levels inside homes were very low, as pets are kept predominantly outdoors [23]. This study has also demonstrated high mite allergen levels in this environment, with 97% of participants having bed mite allergen levels above the proposed threshold for sensitisation (2 μg of Der p 1 per gram of dust) [23]. Allergen levels are unlikely to have changed, and the rise in sensitisation to pet allergens may be explained by an increased population of susceptible children, rather than by an increase in allergen exposure. However, our data also suggest that this increase in susceptibility is not general, but allergen specific (i.e., we observed no time-related trend in mite sensitisation).

In Ghana, firewood and charcoal are commonly used for cooking in rural homes, whereas nonwood fuels (e.g., kerosene, liquefied petroleum gas) are often used in urban homes. Ethiopian studies have reported increased risk of wheeze associated with the use of kerosene [24,25]. However, in our recent study the type of domestic fuel was not associated with childhood asthma [21].

In 1993 we failed to show a significant association between sensitisation and EIB. In contrast, in 2003 sensitisation was significantly associated with an increased risk of EIB. This is likely a result of the larger number of subjects in 2003, although we cannot exclude the possibility that a change has occurred in the relationship between these two phenotypes over the ten-year period. However, the results of the multivariate analysis suggest that the increase in EIB may not be caused by the increase in sensitisation.

### Unanswered Questions

In developing countries improved nutritional status is often a result of improved socioeconomic status, and is usually associated with a more westernised lifestyle. The observation of contemporaneous increase in BMI, atopic sensitisation, and EIB lends support to the notion that the adoption of westernised lifestyles is an important factor in the development of asthma in children developing countries. However, which particular aspects of westernised lifestyles contribute to the development of atopy and asthma in children remain unclear. The possible role of infections in the development of asthma in this community needs further study as well. Other factors such as immunisations and breast-feeding may also be important, but have not as yet been investigated within this setting.

## Abbreviations

BMI: body mass index
CI: confidence interval
EIB: exercise-induced bronchospasm
PEFR: peak expiratory flow rate
R: rural
UP: urban poor
UR: urban rich
